# A case of lupus miliaris disseminatus faciei

**DOI:** 10.1002/ski2.285

**Published:** 2023-09-13

**Authors:** Ruiping Liu, Bin Lu

**Affiliations:** ^1^ Jining Medical University Jining China; ^2^ Affiliated Hospital of Jining Medical University Jining China

## Abstract

We present a case of a 25‐year‐old male patient with lupus miliaris disseminatus faciei, a rare and unexplained skin disease characterised by asymptomatic facial papules. The clinical presentation and histopathological images provided in our study can be used to visualise the features and progression of the disease.

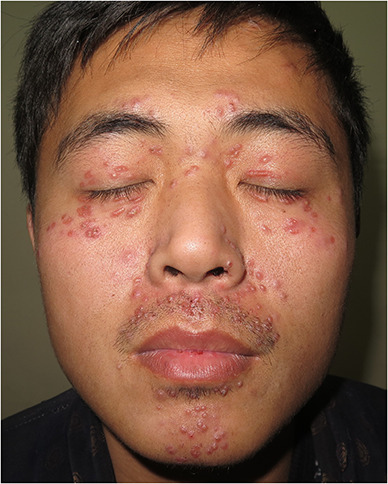

A 25‐year‐old male patient with a 4‐month history of asymptomatic facial papules. He presented with millet‐sized red papules around his eyelids and lips. Some of these papules displayed yellow‐white pustules, while others had already ulcerated and formed scabs (Figure 1a). Histopathological examination showed superficial granulomatous inflammation with perifollicular necrotising granulomas (Figure 1b). The granulomas consist of epithelioid macrophages with multinucleated giant cells, and surrounding lymphocytes (Figure 1c). Based on relevant examinations, a diagnosis of lupus miliaris disseminatus faciei has been made, a condition for which the aetiology and pathogenesis are still not fully understood.^1^

**FIGURE 1:**
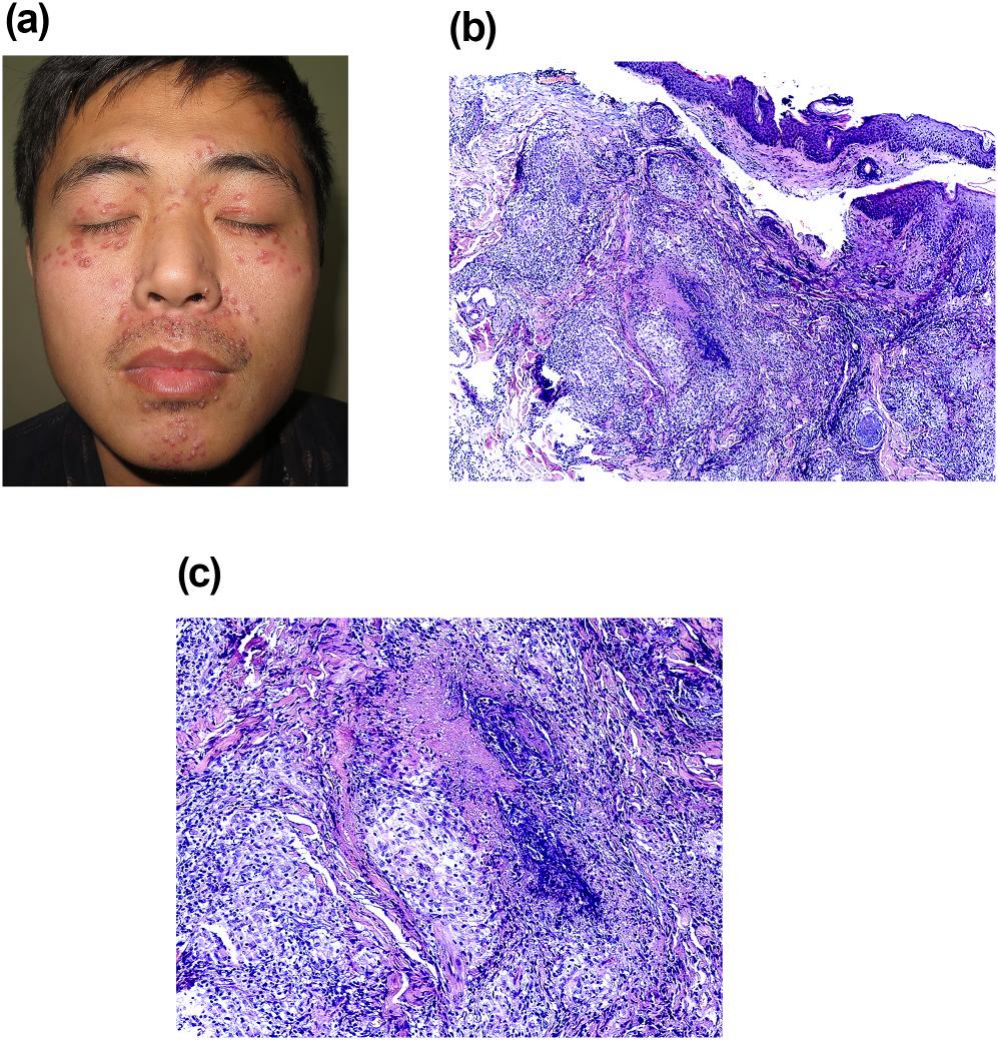
(a) Millet‐sized red papules mainly around the eyelids and lips. (b) Superficial granulomatous inflammation with perifollicular necrotising granulomas. (c) Granulomas with epithelioid macrophages and surrounding lymphocytes.

## CONFLICT OF INTEREST STATEMENT

None to declare.

## AUTHOR CONTRIBUTIONS

**Ruiping Liu**: Writing—original draft (equal); writing—review & editing (equal). **Bin Lu**: Writing—original draft (equal); writing—review & editing (equal).

## ETHICS STATEMENT

This study was conducted in compliance with the ethical principles outlined in the Declaration of Helsinki. All participants provided written informed consent prior to participation.

## Data Availability

The data underlying this article will be shared on reasonable request to the corresponding author.
